# Editorial: Aquatic animal welfare as a driver for the improvement of food production systems

**DOI:** 10.3389/fvets.2023.1297306

**Published:** 2023-11-06

**Authors:** Murilo Henrique Quintiliano, Caroline M. Maia, Ana Silvia Pedrazzani, Carla Forte Maiolino Molento, Joao L. Saraiva

**Affiliations:** ^1^FAI Farms Ltd., Oxford, United Kingdom; ^2^FishEthoGroup Association, Olhão, Portugal; ^3^Alianima Organization, São Paulo, Brazil; ^4^CAUNESP - Centro de Aquicultura da UNESP, Jaboticabal, Brazil; ^5^Wai Ora Aquaculture Environmental Technology Ltd., Curitiba, Brazil; ^6^Animal Welfare Laboratory, Federal University of Parana, Curitiba, Paraná, Brazil; ^7^Fish Ethology and Welfare Group, Centro de Ciências do Mar, Faro, Portugal

**Keywords:** fish welfare, fish stress, welfare indicators, welfare assessment, fish stocking density, enrichment, certification schemes

The aquaculture industry is continuously growing over the years. This dynamic increase has presented with both opportunities and challenges. As the world increasingly turns to aquatic species as a source of high-quality protein, the welfare of these animals remains largely overlooked. Whereas assuring better welfare for farmed aquatic species clearly benefits the animals, it may also result in higher product quality and ultimately in economic gains that are frequently unknown by the producers, retailers, consumers, and other stakeholders. Thus, addressing the welfare issues of fish and other aquatic species around the world becomes increasingly essential. The importance of reliable scientific knowledge to reach this goal must not be underestimated. In this context, the curation of contemporary scientific literature in this field becomes a vital instrument to increase the significance of animal welfare as a necessary topic in the progress of fish farming to a more sustainable food production system.

In this direction, it is important to learn from experience and reduce the missteps witnessed in terrestrial animal farming. In this Research Topic we endeavor to channel scientific expertise to improve the lives of aquatic animals reared under farming conditions. Our objective is clear—to promote animal welfare science applied to aquatic animal life under farming conditions as an important tool to reach better aquaculture systems. This initiative encompasses farmed animals, while also considering the intricate relationships between humans, animals and the environment. By integrating such considerations, we aim to foster a sense of social responsibility and demonstrate that careful attention to the welfare of farmed aquatic animals' yields benefits for the animals that also cascade through producers, consumers, and other stakeholders.

The selection of articles shown here was made to cover key topics involving the improvement of the life quality of farmed aquatic species. Alongside other indicators, stocking densities are a critical point of welfare in aquaculture (Saraiva et al.). High stocking densities may cause stress due to lack of space and an increased competition for food, besides a higher risk of injuries and even diseases. On the other hand, very low densities may cause health problems, stress for social species and more aggressive interactions for territorial species—while possibly hindering the economic viability of fish farms. In this sense, Saraiva et al. brought a deep and well based discussion about the relationship between stocking density and fish welfare. This review shows fundamental insights, highlighting that while precision is achievable through accurate biomass control and water quality assessment, defining strict boundaries for protective stocking density levels remains elusive. The authors advocate an approach combining a robust welfare assessment founded on operational indicators with judicious management practices rather than prescribing rigid stocking limits.

Stocking densities clearly affect the social context in the environment, which was the central theme of another review published in this Research Topic. Cavallino et al. navigate through the intricate connections among cortisol, sex steroids, and social behaviors in teleost fish. By highlighting the interplay between endocrine landscapes and behavioral traits, this review highlights the path toward welfare assessment frameworks rooted in intrinsic biological characteristics, focusing on proposing social behaviors as important welfare indicators in teleost fish. The authors emphasize that reproductive and agonistic behavioral traits such as aggression, anxiety and courtship are fundamental social indicators to assess fish welfare under captive conditions (Cavallino et al.). In addition, whereas physical enrichment has emerged as lead candidate for the application of enrichment strategies within aquaculture settings, there is scarce knowledge about social enrichment for fish. Thus, on a related paper, Zhang et al. bring up a discussion around the effects of social enrichment on growth performance and aggression of black rockfish (*Sebastes schlegelii*) and fat greenling (*Hexagrammos otakii*), which naturally inhabit the same area but are usually farmed in monocultures. By demonstrating that such effects are species-dependent, the same authors highlight that optimizing the welfare of multi-species farming should rely on proper scientific validation regarding the combination of species, the interaction between social and physical enrichment and adequate mitigation measures against potential adverse effects (Zhang et al.).

Stress response due to handling and other common farming procedures is another critical point of fish welfare in aquaculture systems. As for any animal, the slaughtering process is one of the most powerful stressors for farmed fish. In this line, Fiordelmondo et al. investigated the physiological responses of rainbow trout (*Oncorhynchus mykiss*) to pre-slaughtering procedures in a short case study. Through an in-depth analysis of gene expression patterns, plasma analyses, and skin mucus activity, this study offers valuable insights into the influence of management practices on stress levels and welfare indicators for trout during the slaughtering process (Fiordelmondo et al.).

Finally, as the welfare of aquatic farmed species must not be relegated to the periphery of aquaculture development, Browning presents a comprehensive exploration of four current certification schemes and assessment frameworks, also identifying their limitations. Recommendations for a best-practice welfare assessment approach for aquaculture are proposed, such as completeness, validation, feasibility, transparency, and setting thresholds. The author emphasizes that any aquaculture system should be assessed according to suitable frameworks, suggesting the inclusion of feelings-based approaches in addition to the current focus on health. Addressing fish conditions in aquaculture systems must be integral from the outset, since retrofitting welfare considerations becomes extremely complex as systems mature (Browning).

In summary, the confluence of science and practice presents an auspicious avenue for the evolution of aquaculture, especially when it comes to major issues as stocking densities, social contexts and stress responses, as explored in this Research Topic. The current compilation is an example of the progress made in animal welfare within aquatic contexts, while also shedding light on the pressing challenges that demand innovative solutions. We extend our profound gratitude to the thinkers, practitioners and researchers—especially the ones who contributed to this Research Topic—who dedicate themselves to the cause of aquatic animal welfare. We ardently anticipate the transformative contributions that lie on the horizon.

## Author contributions

MQ: Writing—original draft, Writing—review and editing, Conceptualization, Project administration. CMa: Writing—review and editing. AP: Writing—review and editing. CMo: Writing—original draft. JS: Writing—review and editing.

